# Transpapillary biliary biopsy for malignant biliary strictures: comparison between cholangiocarcinoma and pancreatic cancer

**DOI:** 10.1186/s12957-016-0883-8

**Published:** 2016-05-04

**Authors:** Wei-Ming Chen, Kuo-Liang Wei, Yi-Shing Chen, Pey-Jium Chang, Shui-Yi Tung, Te-Sheng Chang, Hao-Chun Huang, Chein-Heng Shen, Yung-Yu Hsieh, Cheng-Shyong Wu

**Affiliations:** Division of Gastroenterology and Hepatology, Department of Internal Medicine, Chang Gung Memorial Hospital, 6 Section West, Chia-Po Road, Putz City, Chiayi 613 Taiwan; Graduate Institute of Clinical Medical Sciences, College of Medicine, Chang Gung University, Taoyuan, Taiwan; College of Medicine, Chang Gung University, Taoyuan, Taiwan

**Keywords:** Biliary biopsy, Biliary stricture, Cholangiocarcinoma, Pancreatic cancer

## Abstract

**Background:**

Tissue sampling for biliary stricture is important for differential diagnosis and further treatment. This study aims to assess the differences of transpapillary biliary biopsy for malignant biliary strictures between cholangiocarcinoma and pancreatic cancer.

**Methods:**

From January 2010 to December 2013, we retrospectively studied 79 patients who suffered from biliary strictures and received transpapillary forceps biopsy after sphincterotomy for tissue sampling. The diagnostic sensitivity, specificity, positive predictive value (PPV), and negative predictive value (NPV) of forceps biopsy were calculated in all cases for both cholangiocarcinoma and pancreatic cancer patients. Possible factors that distinguish malignant strictures from benign strictures and which could affect the accuracy of tissue sampling were analyzed.

**Results:**

There are 65 malignant and 14 benign biliary stricture patients enrolled. The malignant group has a significantly higher serum bilirubin level than the benign group, but age, clinical presentation, level of serum carcinoembryonic antigen (CEA), carbohydrate antigen (CA) 19-9, and alkaline phosphatase are not. The sensitivity, specificity, PPV, and NPV of forceps biopsy for biliary stricture are 53.85, 100, 100, and 31.82 %, respectively. The cholangiocarcinoma group has a higher sensitivity (73.53 versus 29.17 %, *p* < 0.001), older age, lower CA 19-9 level, and more common hepatic duct strictures than the pancreatic group. The age, serum CEA, CA 19-9 and the alkaline phosphatase level, serum bilirubin level >10 mg/dL, tissue sampling ≧3 are not significant factors affecting diagnostic accuracy in forceps biopsy for pancreatobiliary strictures. There is neither major bleeding nor perforation in our study.

**Conclusions:**

Transpapillary forceps biopsy of biliary strictures after sphincterotomy for tissue sampling is safe and a significantly higher sensitive method in cholangiocarcinoma but not in pancreatic cancer.

## Background

Biliary stricture may be benign or malignant. The major etiology of a malignant biliary stricture includes a primary tumor or local extension, such as cholangiocarcinoma or pancreatic head cancer [1]. For further chemotherapy or radiotherapy, tissue sampling at endoscopic retrograde cholangiopancreatography (ERCP) by forceps biopsy, brush cytology or fine needle aspiration is important. The biliary forceps biopsy provides a sample of bile duct tissue deep into the epithelium and theoretically avoids inadequate sampling that may occur with brushing [2]. However, transpapillary bile duct biopsy for biliary strictures has a low and wide range of sensitivity, from 52 to 81 %, and it is time-consuming and technically difficult [2, 3].

According to tumor type, some studies indicate that the cancer detection rate of forceps biopsy is higher in cholangiocarcinoma than in pancreatic head cancer [4–6] but not significantly. Higashizawa et al. reported a significantly higher sensitivity of the biopsy for primary bile duct cancer [7]. Conversely, some studies revealed a higher detection rate in pancreatic head cancer [8–10].

To assess further the accuracy of transpapillary bile duct biopsies in pancreatobiliary strictures and to assess the differences of biliary biopsies between cholangiocarcinoma and pancreatic head cancer, we have reviewed our experience over the 4-year period from January 2010 to December 2013.

## Methods

Seventy-nine consecutive patients with strictures of the extrahepatic bile duct, demonstrated on ERCP, were studied retrospectively. For inclusion in the study, patients had to have a definite final benign or malignant diagnosis based either on independent histological sampling or on clinical and radiological follow-up data for at least 6 months. Patients with a lesion of the papilla were excluded. ERCP was performed using a conventional duodenoscope (JF-200 or JF-230; Olympus Optical Co., Tokyo, Japan). Transpapillary biopsy was performed after sphincterotomy with a standard biopsy forceps (FB-26N-1; Olympus, Tokyo, Japan) that was malleable, angle around degree, with fenestrated cup and a metal sheath, and without tooth or needle. After the forceps were introduced via the papilla into the bile duct and advanced to the biliary lesion, tissue samples (1–8 specimens; mean 4.6) were obtained. Biopsy specimens were fixed in 10 % formalin, embedded in paraffin, and stained with hematoxylin and eosin. A histological result showing atypical cells only in patients with malignant strictures was estimated as negative for cancer.

Statistical analysis was performed using SPSS software version 17.0 (SPSS Inc., Chicago, IL). Patient characteristics were represented as the mean ± standard deviation. Diagnostic sensitivity, specificity, positive predictive value (PPV), and negative predictive value (NPV) of forceps biopsy were calculated in all cases for both cholangiocarcinoma and pancreatic cancer patients. Probable associated factors of positive results including gender, age, chief complication and level of carcinoembryonic antigen (CEA), carbohydrate antigen 19-9 (CA 19-9), alkaline phosphatase, total bilirubin, and the numbers of specimens were assessed. The diagnosis of cholangitis was based on the Tokyo guidelines [11]. Statistical correlations were considered significant for *p* < 0.05. Ethics approval for the chart review was performed through the Chang Gung Medical Foundation Institutional Review Board (no. 101-3175B).

## Results

### Patients’ characteristics

Most patients were males, presenting painless jaundice and had common bile duct (CBD) stricture (Table 1). There were no statistically significant differences between benign and malignant groups with respect to the clinical presentation, level of serum CEA, CA 19-9, alkaline phosphatase, or level of biliary stricture. Patients suffered from more cholangitis but less painless jaundice in the benign than malignant group, proportionately. The only significant factor that differentiates the benign from the malignant stricture is the level of serum total bilirubin in our study.

**Table 1:** Characteristics of patients in forceps biopsy for biliary stricture

	Total (*n* = 79)	Malignant (*n* = 65)	Benign (*n* = 14)	*P* ^a^
Age (years)	68.59 ± 12.02	69.15 ± 11.15	66.00 ± 15.66	0.376
Sex (male to female)	53:26	44:21	9:5	0.806
Chief complaint				
Jaundice	52 (66 %)	44 (68 %)	8 (57 %)	0.450
Pain	3 (4 %)	2 (3 %)	1 (7 %)	0.470
Cholangitis	19 (24 %)	15 (19 %)	4 (29 %)	0.663
Stent exchange	2 (3 %)	2 (3 %)	0 (0 %)	0.506
CBD dilatation	2 (3 %)	1 (2 %)	1 (7 %)	0.226
Pancreatitis	1 (1 %)	1 (2 %)	0 (0 %)	0.640
Total bilirubin (mg/dL)	8.43 ± 6.33	9.14 ± 6.47	4.87 ± 4.18	0.025*
CEA (ng/mL)	18.90 ± 57.73	20.58 ± 60.95	6.08 ± 9.78	0.566
CA 19-9 (U/mL)	1863.70 ± 5062.73	2122.58 ± 5736.81	88.54 ± 75.95	0.325
ALKP (U/L)	365.65 ± 320.33	367.86 ± 322.48	352.80 ± 324.09	0.892
Stricture level (CBD:CHD)	54:25	42:23	12:2	0.124

*Abbreviations*: *CEA* carcinoembryonic antigen, *CA 19-9* carbohydrate antigen 19-9, *ALKP* alkaline phosphatase, *CBD* common bile duct, *CHD* common hepatic duct

^a^Comparison between malignant and benign group

*P<0.05

Comparing the patients’ characteristics between the cholangiocarcinoma and pancreatic cancer group, these two groups were similar in the male-to-female ratio, level of serum total bilirubin, CEA, alkaline phosphatase, and number of biopsy specimens (Table 2). In the pancreatic cancer group, patients were characteristically of a younger age, higher level of serum CA 19-9, and all suffering from CBD strictures.

**Table 2:** Characteristics of patients between cholangiocarcinoma and pancreatic head cancer

	Cholangiocarcinoma (*n* = 34)	Pancreatic head (*n* = 24)	*P*
Age (years)	71.97 ± 9.72	65.38 ± 10.90	0.019*
Sex (male to female)	20:14	19:5	0.104
Total bilirubin (mg/dL)	9.55 ± 6.57	8.49 ± 6.68	0.551
CEA (ng/mL)	25.61 ± 80.56	9.49 ± 17.80	0.369
CA 19-9 (U/mL)	411.09 ± 992.54	4441.51 ± 7462.84	0.017*
Alkaline phosphatase (U/L)	359.34 ± 338.81	269.43 ± 137.32	0.254
Stricture level (CBD:CHD)	15:19	24:0	<0.001*
Numbers of specimens	4.61 ± 1.52	4.55 ± 1.77	0.895
Specimens <5: ≥5	17:17	9:15	0.585

*CEA* carcinoembryonic antigen, *CA 19-9* carbohydrate antigen 19-9

*P<0.05

### Correlation between biopsy results and final diagnosis

Table 3 summarizes the correlation between the biopsy result and the final clinicopathological diagnoses for the 79 patients. There were no false positives in the benign biliary strictures. Most malignant strictures were caused by cholangiocarcinoma (52.31 %) and pancreatic head cancer (36.92 %). We did re-biopsies on the malignant strictures where previous biopsies had showed atypical results in the cholangiocarcinoma and pancreatic cancer groups. There were four positive malignant findings (80 %) in the cholangiocarcinoma group but none in the pancreatic head group.

**Table 3:** Correlation between biopsy result and the final clinicopathological diagnosis

Clinicopathological diagnosis	Biopsy result		
	Negative	Atypia	Malignant
Benign (*n* = 14)	14	0	0
Malignant (*n* = 65)			
Cholangiocarcinoma (*n* = 34)	4	5	25
Pancreatic head cancer (*n* = 24)	15	2	7
Colon cancer (*n* = 2)	2	0	0
Hepatocellular carcinoma (*n* = 3)	0	0	3
Gallbladder cancer (*n* = 1)	0	1	0
Lung cancer with liver metastasis (*n* = 1)	0	1	0
Total (*n* = 79)	35	9	35

### Diagnostic performance of biliary biopsy

The sensitivity of forceps biopsies for malignant biliary strictures in all patients, cholangiocarcinoma, pancreatic head cancer, and other groups is 53.85, 73.53, 29.17, and 42.86 %, respectively. The sensitivity is significantly higher in the cholangiocarcinoma group than in the pancreatic cancer group. All groups showed perfect specificity and positive predictive values. However, the negative predictive value was low in these four groups (Table 4).

**Table 4:** Diagnostic performance of biliary biopsy for diagnosing malignant biliary stricture

Malignancy	Total	CCC	Pancreas	Others^a^
Sensitivity (%)	53.85	73.53	29.17	42.86
Specificity (%)	100.00	100.00	100.00	100.00
PPV (%)	100.00	100.00	100.00	100.00
NPV (%)	31.82	60.87	45.16	77.78

CCC cholangiocarcinoma, *PPV* positive predictive value, *NPV* negative predictive value

^a^Others include colon cancer, hepatocellular carcinoma, gallbladder cancer, and lung cancer with liver metastasis

### Factors in predicting positive biopsy results

The possible factors associated with positive malignant findings in tissue sampling by forceps biopsy included stricture level, level of serum CEA, CA 19-9 and alkaline phosphatase, serum bilirubin >10 mg/dL, numbers of biopsy specimens, biopsy specimens ≧5, and biopsy specimens ≧3 revealed no statistically significant results in all malignant, cholangiocarcinoma, and pancreatic head cancer groups. Regarding the level of stricture, the biopsy from common hepatic duct (CHD) tended to have positive results when compared with CBD (69.57 versus 45.24 %, odds ratio 2.767; 95 % CI, 0.943–8.116; *P* = 0.06) in malignant biliary stricture but not in the cholangiocarcinoma group.

### Complications

Regarding accidents related to forceps biopsy, there is no perforation of the bile duct occurring. Hemobilia was found during the examination after forceps biopsy in all patients, but it subsided spontaneously and no further therapeutic endoscopy was needed. There was one patient who is suffering from pancreatitis after ERCP (1.27 %) and recovered after conservative treatment. We performed a sphincterotomy to all patients, but no accidents related to the sphincterotomy occurred.

## Discussion

Although multiple-row detector computerized tomography (CT) and magnetic resonance cholangiopancreatography (MRCP) make the diagnosis of biliary strictures more comprehensive and reliable, the differential diagnosis between malignant and benign biliary strictures is still difficult for small lesions [12] or primary sclerosing cholangitis (PSC) predisposed to malignancy [13]. Clinical features or laboratory tests alone usually cannot differentiate malignant from benign proximal bile duct strictures. From previous studies, plasma transaminase values were significantly elevated in the malignant group, but bilirubin, alkaline phosphatase, and lactate dehydrogenase (LDH) failed to identify patients with a malignancy [14]. In primary sclerosing cholangitis patients, a combination of abnormal CEA (>5.2 ng/mL) and CA 19-9 (>180 U/mL) have 100 % sensitivity to the diagnosis of bile duct malignancy [15], but this result was not suggested as a diagnostic criteria [16]. In our study, most patients who suffered from malignant biliary strictures were presenting painless jaundice, a higher level of serum CEA and CA 19-9 but not statistically significant. However, a wide range of our data and small sample size might limit our results. Not the same as the previous study [14], the level of serum total bilirubin was significantly higher in the malignant group in our study.

Tissue sampling of malignant biliary strictures via the endoscopic or percutaneous approach is essential for further surgery or chemotherapy. The endoscopic approach for tissue sampling of suspected bile duct tumors can be obtained by forceps biopsy, brush cytology, endoscopic ultrasound (EUS)-guided fine needle aspiration (FNA), and cholangioscopy-directed biopsy [17]. Forceps biopsy for malignant biliary strictures is commonly used in clinical practice with a higher sensitivity compared to brush cytology alone in some studies [9, 18]. However, forceps biopsy is less routinely used for biliary sampling than brush cytology because it is technically challenging and has increased procedure time [9, 10]. In our study, the sensitivity and specificity of forceps biopsy for biliary strictures are 53.85 and 100 %, respectively, which is similar to the previous studies. Technical improvements in both accessories and endoscopes increase the detection rate of cancer by forceps biopsy. Sugiyama et al. designed a new malleable forceps biopsy that could be introduced into the bile duct without sphincterotomy [5]. The Howell Introducer is a new instrument developed to easily collect multiple tissue samples for forceps biopsy and brush cytology, which increases the sample collection rate [10, 19]. Tamada et al. report that a new ropeway-type biopsy forceps is useful for selectively obtaining biopsy specimens of the bile duct [20]. Large-capacity forceps have better adequacy of the sample, submucosal sampling, and cancer detection rate than the standard forceps [21, 22]. Forceps biopsy with a combination of intraductal ultrasonography (IDUS) compensates for the false-negative rate [6]. Brush cytology is the most frequently used tissue sampling technique and is technically easy, less time-consuming, and generally safe. Its sensitivity for cholangiocarcinoma is higher than in pancreatic cancer, which ranges from 23 to 80 % and 0 to 66 %, respectively [3]. Digitized image analysis (DIA) and fluorescence in situ hybridization (FISH) have been used to increase the sensitivity compared to routine cytology [23, 24]. A combination of the forceps biopsy and brush cytology increases the sensitivity by approximately 15 to 25 % compared with either method alone [8, 9, 18]. EUS provides real-time imaging of the target lesion and adjacent organs and obtains tissue through FNA of visualized masses with reported sensitivity for malignancy of 43 to 86 % [25–27]. Cholangioscopy allows for a direct visualization of the biliary tree and performs direct tissue sampling. Shah et al. report that the sensitivity and specificity to detect malignancy by cholangioscopy with and without biopsy in patients with indeterminate pancreatobiliary pathology was 89 and 96 %, respectively [28]. The SpyGlass Direct Visualization System for peroral cholangioscopy plus dedicated mini-forceps biopsy (SpyBite; Boston Scientific) has many improvements over previous cholangioscopy platforms [29]. The sensitivity is 71 to 100 % by using the SpyGlass system with SpyBite forceps biopsy in pancreatobiliary lesions [30–32]. Draganov et al. reveal a significantly higher accuracy in cholangioscopy-guided biopsies than ERCP-guided cytologic brushings and standard forceps biopsies [33].

Tamada et al. summarize the sensitivity of transpapillary biliary biopsy between cholangiocarcinoma and pancreatic cancer [3]. The sensitivity of the biopsy is low (33–71 %) if the tumor is outside the bile duct, such as pancreatic cancer. However, the sensitivity of the biopsy is higher (30–89 %) if the tumor is inside the bile duct, such as cholangiocarcinoma. Conversely, some reports indicate that the sensitivity in pancreatic cancer is higher by using transpapillary biliary biopsy but not statistically significantly [8–10]. Higashizawa et al. report on the sensitivity of the biopsy for primary bile duct cancer by using malleable biopsy forceps (FB-39Q; Olympus, Tokyo, Japan) with a Teflon sheath, and guidewire facilitating was significantly higher than pancreatic cancer (83 versus 47 %, *p* < 0.05) [7]. In comparative studies between EUS-guided FNA and transpapillary forceps biopsy plus brush cytology for malignant biliary stricture, the sensitivity was better for ERCP-based techniques in bile duct cancer, whereas EUS-guided FNA was superior for pancreatic cancer [34, 35]. In our study, we used the conventional biopsy forceps (FB-26N-1; Olympus, Tokyo, Japan), which is cheap and widely available in bile duct sampling. Although it was not an innovated study, our data showed that the sensitivity in cholangiocarcinoma was significantly higher than that in pancreatic cancer (73.53 versus 29.17 %, *p* = 0.01) via transpapillary biliary biopsy. Hence, tissue sampling for pancreatic-cancer-related biliary strictures by using the EUS-guided FNA or cholangioscopic approach might be a better method than forceps biopsy.

The choice between repeating sampling and performing additional methods in negative or non-diagnostic results for malignant biliary strictures is still controversial. One study shows a 56 % (10/18) positive rate in repeating brush cytology for pancreatobiliary strictures [36]. Another study finds that repeated brushing significantly improves the sensitivity from 35 to 44 % [37]. DeWitt indicates that EUS-guided FNA of proximal biliary strictures after negative ERCP brush cytology results and reveals a high sensitivity (77 %) but low NPV (29 %) result [38]. Coté and Sherman suggest a cholangioscopic approach in highly suspected malignant PSC patients and EUS-guided FNA or cholangioscopic approach in non-PSC patients with proximal stricture after a negative brush cytology result [39]. Our study shows a high positive rate (80 %) of repeat biopsy in the cholangiocarcinoma group that showed atypical results in previous forceps biopsy. However, this result is not seen in the pancreatic cancer group. Further large case numbers and prospective study is needed to decide on the next step after a negative result of forceps biopsy.

From the literature review, many studies discuss the factors affecting the sensitivity of brush cytology but few about forceps biopsy. The possible factors associated with positive yields of biliary brush cytology include older age, serum bilirubin levels >10 mg/dL, a mass on cross-sectional imaging, mass size >1 cm, and biliary stricture length of >1 cm [40, 41]. Similar to brush cytology, Kimura et al. report that the serum bilirubin level ≧10 mg/dL and ≧3 tissue samplings are independent factors affecting cancer-positive rate in forceps biopsy for pancreatic cancer [42]. In one study, whether the lesion is flat or not significantly affects the accuracy of brush cytology and fluoroscopic forceps biopsy for biliary neoplasm [43]. In our study, the accuracy is higher for forceps biopsy from CHD than CBD. The age, serum CEA, CA 19-9, and alkaline phosphatase level, serum bilirubin level >10 mg/dL, tissue sampling ≧3, tissue sampling ≧5 are not significant factors that affect diagnostic accuracy.

Complications relating to forceps biopsy for tissue sampling of biliary strictures include perforation and hemorrhage. Pugliese et al. report one biliary perforation that happened after forceps biopsy from CHD in a 52-cases study (1.9 %) [44]. Schoefl et al. report one major hemorrhage that needed further endoscopic intervention occurring after biopsy in 119 cases (0.8 %) [18]. In our study, only minor hemorrhaging but no major bleeding nor perforation happened. The major complications after sphincterotomy are similar to forceps biopsy. The complication rate of perforation and hemorrhage is 0.6 and 1.3 % [45], respectively. Although sphincterotomy is not necessary for biliary forceps biopsy by using a specially designed biopsy forceps (FB-39Q; Olympus, Tokyo, Japan) [5], we did a sphincterotomy on all of our patients for easier forceps (FB-26N-1; Olympus, Tokyo, Japan) insertion. There is no major complication that happened.

## Conclusions

Although forceps biopsy for biliary stricture is considered to be time-consuming, difficult, and uncommonly used technique, it is safe and significantly sensitive in cholangiocarcinoma but not in pancreatic cancer for tissue sampling.

## Abbreviations

CA: carbohydrate antigen
CBD: common bile duct
CEA: carcinoembryonic antigen
CHD: common hepatic duct
CT: computerized tomography
DIA: digitized image analysis
ERCP: endoscopic retrograde cholangiopancreatography
EUS: endoscopic ultrasound
FISH: fluorescence in situ hydridization
FNA: fine needle aspiration
IDUS: intraductal ultrasonography
LDH: lactate dehydrogenase
MRCP: magnetic resonance cholangiopancreatography
NPV: negative predictive value
PPV: positive predictive value
PSC: primary sclerosing cholangitis
